# Caspase-1 activity affects AIM2 speck formation/stability through a negative feedback loop

**DOI:** 10.3389/fcimb.2013.00014

**Published:** 2013-04-24

**Authors:** C. Juruj, V. Lelogeais, R. Pierini, M. Perret, B. F. Py, Y. Jamilloux, P. Broz, F. Ader, M. Faure, T. Henry

**Affiliations:** ^1^International Center for Infectiology Research, Université de LyonLyon, France; ^2^INSERM U1111Lyon, France; ^3^CNRSUMR 5308, France; ^4^LabEx Ecofect, Ecole Normale SupérieureLyon, France; ^5^Department of Microbiology and Immunology, School of Medicine, Stanford UniversityStanford, CA, USA

**Keywords:** inflammasome, AIM2, *Francisella tularensis*, regulation, caspase-1

## Abstract

The inflammasome is an innate immune signaling platform leading to caspase-1 activation, maturation of pro-inflammatory cytokines and cell death. Recognition of DNA within the host cytosol induces the formation of a large complex composed of the AIM2 receptor, the ASC adaptor and the caspase-1 effector. *Francisella tularensis*, the agent of tularemia, replicates within the host cytosol. The macrophage cytosolic surveillance system detects *Francisella* through the AIM2 inflammasome. Upon *Francisella novicida* infection, we observed a faster kinetics of AIM2 speck formation in ASC^KO^ and Casp1^KO^ as compared to WT macrophages. This observation was validated by a biochemical approach thus demonstrating for the first time the existence of a negative feedback loop controlled by ASC/caspase-1 that regulates AIM2 complex formation/stability. This regulatory mechanism acted before pyroptosis and required caspase-1 catalytic activity. Our data suggest that sublytic caspase-1 activity could delay the formation of stable AIM2 speck, an inflammasome complex associated with cell death.

## Introduction

The inflammasome is an innate immune signaling platform sensing danger signals and microbial infections leading to caspase-1 activation. Active caspase-1 triggers the maturation of pro-inflammatory cytokines IL-1β and IL-18 and cell death in a process termed pyroptosis (Martinon et al., 2002). Inflammasome activation proceeds via the formation of a multimolecular complex containing a receptor, an adaptor such as ASC and the cysteine protease, caspase-1. AIM2 is an inflammasome receptor detecting DNA within the host cytosol. AIM2 activation has been reported during infection with cytosolic bacteria or DNA viruses (Fernandes-Alnemri et al., 2010; Jones et al., 2010; Rathinam et al., 2010; Sauer et al., 2010) as well as during autoinflammatory syndromes such as psoriasis (Dombrowski et al., 2011). *Francisella tularensis*, the agent of tularemia and its close relative *Francisella novicida*, are Gram-negative bacteria replicating within the macrophage cytosol. Escape from the vacuole into the host cytosol is dependent on a genetic locus, the *Francisella* pathogenicity island (FPI) (Lindgren et al., 2004). Lysis of *F. novicida* and release of the bacterial DNA in the cytosol lead to AIM2 inflammasome activation. The AIM2 inflammasome is required for efficient innate immune responses to *F. novicida* (Fernandes-Alnemri et al., 2010; Jones et al., 2010) and this bacterium is emerging has a key model to study AIM2 inflammasome (Fernandes-Alnemri et al., 2010; Rathinam et al., 2010; Pierini et al., 2012; Puri et al., 2012).

In addition to its key antimicrobial role (Brodsky and Monack, 2009), the inflammasome has been implicated in numerous autoinflammatory syndromes associated with either mutations of receptors such as NLRP3 (also known as cryopyrin) or with the chronic presence of danger signals (Jeru et al., 2008; Dombrowski et al., 2011; Mason et al., 2012). These diseases highlight the need for a tight regulation of this pathway (Rathinam et al., 2012). In this work, using *F. novicida* infection, we identified a novel negative feedback loop regulating the AIM2 inflammasome. We demonstrate that in the absence of a functional AIM2 inflammasome, AIM2/ASC complex forms very rapidly, while detection of AIM2/ASC complex is largely delayed in presence of active caspase-1. Our work thus highlights a novel and original regulatory mechanism that dampens AIM2 inflammasome activation.

## Materials and methods

### Cell lines and bone marrow-derived macrophages

Phoenix-Eco packaging cells and L929-ISRE-luciferase were grown in DMEM medium (Life Technologies) supplemented with 10% fetal calf serum (Lonza). Preparation, culture, infection and transduction of bone marrow macrophages were performed as previously described (Henry et al., 2007). This study was carried out in strict accordance with the French recommendations in the Guide for the ethical evaluation of experiments using laboratory animals (http://gircor.net/qui/ethicalEvaluationGuide4LaboratoryAnimals.pdf) and the European guidelines **86/609/CEE.** Experimental studies using murine bone marrow-derived macrophages were approved by the bioethic committee CECCAPP (protocol #ENS_2009_020). C57BL/6J WT mice were purchased from Charles River laboratories. ASC^KO^ mice (Mariathasan et al., 2004) were obtained from V. Dixit (Genentech, South San Francisco). Casp1^KO^ mice were obtained from D. Monack (Stanford university, Stanford, USA). ASC^KO^ and Casp1^KO^ mice were bred at the PBES animal facility and have been back-crossed to C57BL/6 mice at least 10 times. Retroviral particles were generated in Phoenix-Eco packaging cells using pMSCV2.2-derived plasmids. Plasmids encoding GFP-AIM2 (Pierini et al., 2012), casp1_WT_ and casp1_DEAD_ (Broz et al., 2010) have been described before. In addition to the transgene, the two latter plasmids encode GFP under an IRES sequence. In Casp1^KO^ complementation experiments, transduced macrophages were sorted using an Aria II Cell Sorter (BD Biosciences) at day 7 post-isolation. Transduced macrophages were infected at day 9 post-isolation.

### P(dA:dT) transfection and nigericin assay

For prestimulation, BMM were treated overnight with 100 ng/mL LPS (Sigma) or 100 ng/mL Pam3CSK4 (Invivogen). Cell transfection was performed by using lipofectamine 2000 (Life Technologies) following manufacturer's instructions. 0.05–1 μg of poly(dA-dT)_poly(dT-dA) [p(dA:dT)] (Invivogen) were used to transfect 10^5^ BMM. For nigericin assay, cells were prestimulated with LPS (100 ng/ml for 16 h) and treated with 10 μM nigericin (Sigma) for the indicated time.

### Bacterial strains, infections and intracellular replication assay

*Francisella novicida* strain Utah (U112) and an isogenic mutant lacking the whole *Francisella* pathogenicity island (ΔFPI) were grown in tryptic soy broth (TSB) supplemented with 0.1% (w/v) cysteine. BMM were infected as described before (Pierini et al., 2012). Macrophages were lysed with 1% (w/v) saponin (Sigma) in water for 5 min and plated on TSA supplemented with 0.1% (w/v) cysteine to enumerate Colony-forming units (CFU). Analysis of intracellular bacterial growth was also performed using a CantoII FACS (BD Biosciences) by detecting the fluorescence emission of intracellular U112 harboring the pKK-214-GFP plasmid. When applicable, IFN-β (100 u/ml at 3 h BI, R&D systems), glycine (25mM at 1 h PI, Sigma) or z-YVAD-FMK (50 μM at 1 h PI, Bachem) was added. *L. pneumophila* wild-type JR32 strain (WT) was cultured for 48 h on buffered charcoal-yeast extract agar plates (BCYE). For infection experiment, bacteria were grown in ACES-buffered yeast extract medium overnight and diluted in macrophage medium to obtain a MOI of 10. Infections were carried out by centrifuging bacteria onto BMM at 200 g for 10 min. Thirty minutes post infection, cells were washed and fresh medium added.

### Immunofluorescence microscopy

Cells were stained with either rabbit anti-mouse AIM2 (Genentech) at 1/500 dilution, rabbit anti mouse ASC (Santa Cruz), rat anti mouse ASC (Genentech) or a chicken anti-*Francisella* antibody at 1/1000 dilution. For cell death assay, BMM were incubated with EthD-1 at 1 μ M final for 15 min at 37°C before fixation with 4% PFA. Three biological replicates were made per investigated condition. For each coverslip, speck-forming cells or EthDi^+^ cells were counted in ten independent fields representing more than 200 cells using a Nikon Eclipse inverted fluorescence microscope.

### FACS assay

FACS staining was performed on adherent and floating cells. Cells were stained with propidium iodide (5 μg/ml) before analysis with a Canto II FACS (BD Biosciences). Quantification of vacuolar escape using the β-lactamase-CCF4 assay (Life technologies) was performed following manufacturer's instructions. Briefly, macrophages were infected as previously described for 1 or 2 h, washed and incubated in CCF4 for 1h at room temperature in the presence of 2.5 mM probenicid (Sigma). Propidium iodide negative cells were considered for the quantification of cells containing cytosolic *F. novicida* using excitation at 405 nm and detection at 450 nm (cleaved CCF4) or 535 nm (intact CCF4).

### Elisa and LDH assays

DuoSet ELISA kits for IL1-β and TNF-α Elisa (R&D Systems) were performed following manufacturer's instructions. LDH release was measured using the CytoTox96 LDH kit (Promega), following manufacturer's instructions. Cell death was calculated as follow: (sample value − uninfected sample value)/(triton X100-treated sample value − uninfected sample value) ×100.

### Interferon assay

L929 ISRE-luciferase cells were plated the day before at 10^5^ cells per well in a 96 wells-plate. Supernatants from infected BMM were added for 4 h onto the ISRE-luciferase cells. Luciferase luminescence was detected using Bright Glo Assay (Promega) following manufacturer's instructions.

### Western blotting

Cells were lysed in lysis buffer (10mM HEPES-KOH, pH 7.5, 2mM EDTA, 250mM sucrose, 0.1% CHAPS) supplemented with complete protease inhibitor cocktail (Roche). For immunodetection of caspase-1 p10, BMM were infected with U112 and placed in phenol red minus DMEM without MCS-F or SVF. Supernatants were precipitated with 10% trichloroacetic acid (TCA). The obtained pellet was washed twice in cold acetone and resuspended in SDS-PAGE loading buffer. For each sample, the cell lysates and the supernatant corresponding to 2 × 10^6^ BMM were loaded in individual lanes. Rabbit anti-ASC (Santa Cruz Biotechnology), rabbit anti-caspase-1 p10 (Santa Cruz Biotechnology), rabbit anti-LC3 (Sigma), mouse anti-caspase-1 p20 (Adipogen) and mouse anti-actin (Sigma) were used at a 1/1000, 1/500, 1/1000, 1/1000, and a 1/2000 dilution, respectively. Pyroptosome enrichment and ASC cross-linking protocols were adapted from Fernandes-Alnemri and Alnemri (2008) using 10^7^ BMM per sample.

## Results

### AIM2 specks are observed earlier and with a greater frequency in Casp1^KO^ and ASC^KO^ macrophages as compared to WT macrophages

**Figure 1:**
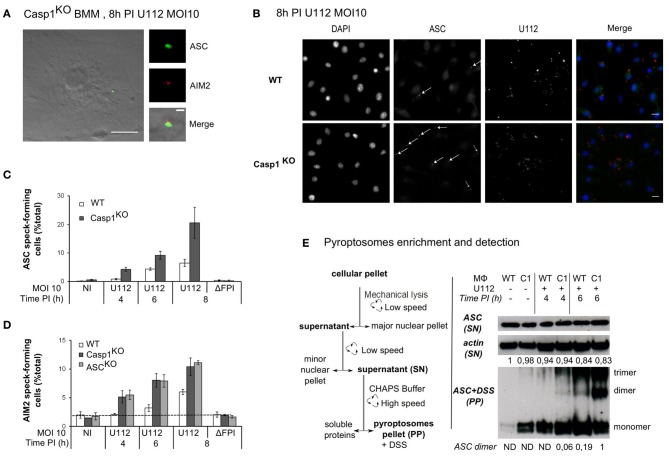
**ASC/caspase-1 negatively regulates AIM2 speck formation during *Francisella* infection. (A–E)** BMM from the indicated genotypes were infected with *F. novicida* or the ΔFPI mutant at a MOI of 10 for the indicated time. **(A)** Endogenous ASC (green) and AIM2 (red), **(B)** endogenous ASC (green) and U112 bacteria (red) were detected by immunofluorescence (scale bar: 10 μm). Representative images are shown. **(A)** The inset corresponds to a magnification of the AIM2/ASC speck (scale bar: 1 μm). ASC **(C)** and AIM2 **(D)** speck-forming cells were scored by immunofluorescence microscopy. **(E)** The pyroptosome enrichment protocol (left panel) and the immunoblots of β-actin and endogenous ASC before and after chemical cross-linking (right panel) are shown. Bands quantification was performed using ImageJ software. The numbers below each lane indicate the amount of β-actin (top panel) or ASC dimers (lower panel), ND, not detectable. One experiment representative of two **(D)** or more than two **(C,E)** independent experiments is shown (mean and standard deviation of triplicates are shown).

As previously described (Fernandes-Alnemri et al., 2010; Jones et al., 2010; Rathinam et al., 2010), infection of bone marrow derived macrophages with *F. novicida* led to activation of the AIM2 inflammasome and formation of a large complex, which included AIM2 and ASC (Figure 1A). This complex visible by immunostaining of endogenous AIM2 or ASC proteins is termed AIM2/ASC speck and is considered as the platform on which the mature inflammasome (containing caspase-1) is built (Jones et al., 2010; Jin et al., 2012). In contrast to the pyroptosome, which has been described for the NLRP3 inflammasome as a complex lacking the inflammasome receptor (Fernandes-Alnemri et al., 2007; Fernandes-Alnemri and Alnemri, 2008), the AIM2/ASC speck contains both the inflammasome receptor and the adaptor (Jones et al., 2010). Most assays to monitor inflammasome activation rely on the detection of caspase-1 cleavage products or of caspase-1 substrates (e.g., IL-1β). Quantification of AIM2/ASC speck numbers by immunofluorescence microscopy gave us the opportunity to monitor activation of the pathway at the level of the receptor and/or the adaptor even in macrophages deficient for caspase-1. To investigate the role of the inflammatory caspases, caspase-1, and caspase-11 in AIM2/ASC specks formation, we infected caspase-1/caspase-11-deficient macrophages (Kayagaki et al., 2011) (hereafter termed Casp1^KO^ macrophages) with *F. novicida* at a low MOI (10:1). In agreement with Jones et al. (Jones et al., 2010), we observed the presence of AIM2 and ASC specks in Casp1^KO^ macrophages (Figure 1A). While the physiological relevance of AIM2/ASC specks in absence of caspase-1 remains unclear, this observation indicated that caspase-1 and caspase-11 were not required for the formation of AIM2/ASC specks. Surprisingly, when observing *F. novicida*-infected macrophages at early time points post-infection (PI), we noticed a greater number of ASC specks in Casp1^KO^ as compared to WT macrophages (Figure 1B). A detailed analysis of the kinetics of ASC speck formation revealed that ASC speck could be detected in *F. novicida*-infected Casp1^KO^ macrophages as early as 4 h PI (Figure 1C). In contrast, no ASC specks were visible in WT macrophages at this time point. This result indicated that, upon *F. novicida* infection, WT macrophages showed a delay in ASC speck formation as compared to Casp1^KO^ macrophages or that specks formed in WT macrophages were too unstable to be visible by immunofluorescence. This phenotype was still present at 6 h PI. At this time, while the number of ASC speck-containing cells was relatively low due to the early time PI and the low MOI, ASC specks-containing Casp1^KO^ macrophages were twice as numerous as ASC specks-containing WT macrophages. We did not observe any ASC specks in Casp1^KO^ macrophages infected with a ΔFPI *F. novicida* mutant (data not shown) indicating that ASC speck formation in Casp1^KO^ macrophages, although faster, still required the ability of *F. novicida* to escape into the host cytosol. To investigate if the delay in ASC speck formation observed in WT macrophages was due to a delay in AIM2 speck formation, we analyzed the kinetics of AIM2 speck formation in WT, Casp1^KO^ and ASC^KO^ macrophages (Figure 1D). Upon infection, as observed with ASC specks, AIM2 specks were detectable 2 h earlier in Casp1^KO^ than in WT macrophages. Furthermore, the accelerated kinetics was observed in both Casp1^KO^ and ASC^KO^ macrophages. As these two macrophage lines are deficient for inflammasome activation in response to *F. novicida*, these results suggested that caspase-1 activation was required to delay AIM2/ASC speck formation in WT macrophages infected with *F. novicida*. To confirm this microscopy-based result, we applied a protocol of partial purification (Fernandes-Alnemri and Alnemri, 2008) of the ASC pyroptosomes in infected WT and Casp1^KO^ macrophages and investigated the oligomeric state of ASC using a chemical cross-linker (DSS) as previously described (Fernandes-Alnemri and Alnemri, 2008; Figure 1E). Consistently with the accelerated kinetics of AIM2/ASC speck formation observed by immunofluorescence, we detected ASC dimers and trimers 2 h earlier in Casp1^KO^ macrophages than in WT macrophages (Figure 1E). ASC dimers were detected in WT macrophages 6 h PI in at least five fold lower quantity as compared with Casp1^KO^ macrophages at this same time point. Altogether, these results highlight the presence of a caspase-1-dependent pathway that negatively regulates AIM2/ASC complex formation or stability.

**Figure 2:**
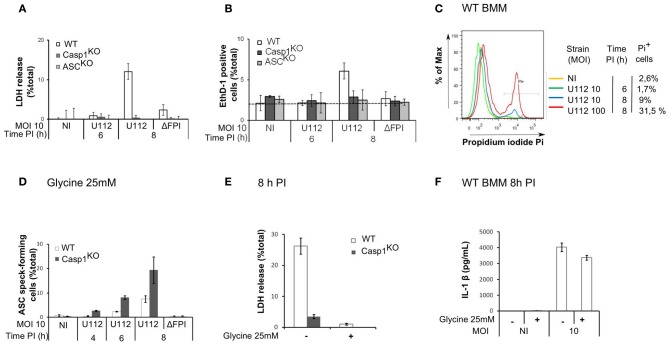
**The ASC/caspase-1-mediated negative regulation of AIM2 complex formation is not due to an early death of WT macrophages. (A–C)** Kinetics of cell death in BMM from the indicated genotypes infected with *F. novicida* or the ΔFPI mutant at the indicated MOI for the indicated time. Cell death was determined by LDH assay **(A)**, microscopy following EthD-1 addition **(B)** and flow cytometry using propidium iodide **(C)**. **(D–F)** BMM were treated with glycine (25mM) at 1 h PI. ASC speck-forming cells were scored by immunofluorescence microscopy at the indicated time post-infection **(D)**. Cell death **(E)** and IL-1β in the supernatant **(F)** at 8 h PI were quantified by LDH assay and ELISA, respectively. One experiment representative of three independent experiments **(A–F)** is shown (mean and standard deviation of triplicates are shown).

Since the AIM2 inflammasome triggers pyroptosis of *F. novicida*-infected macrophages (Mariathasan et al., 2005; Fernandes-Alnemri et al., 2010; Jones et al., 2010), we investigated whether the delay in AIM2/ASC speck formation was due to an early death of WT macrophages. While we observed AIM2/ASC speck formation in Casp1^KO^ macrophages as early as 4 h PI (Figure 1B), at the low MOI used in this study, we could not detect any death in WT macrophages before 8 h PI (Figures 2A–C). The absence of death before 8 h PI was validated by three different assays (LDH release assay-Figure 2A, microscopy analysis using the membrane-impermeant dye, Ethidium homodimer-Figure 2B; Flow cytometry analysis of propidium iodide positive cells-Figure 2C). This death kinetics was similar to the one obtained previously using real time monitoring of pore formation (Pierini et al., 2012). These results strongly suggested that the delay in the formation of AIM2/ASC speck in WT macrophages was not caused by an early WT macrophage death. To formerly exclude death of WT macrophages as a confounding factor, we determined the kinetics of ASC speck formation in presence of extracellular glycine, which blocks caspase-1-mediated pyroptosis (Fink and Cookson, 2006). Importantly, even in absence of macrophage death (Figure 2E), WT macrophages still displayed a delay in ASC speck formation as compared to Casp1^KO^ macrophages (Figure 2D). As previously described for *Salmonella* (Fink and Cookson, 2006), glycine inhibited *Francisella*-mediated pyroptosis without blocking IL-1β release (Figure 2F). Taken together, these results demonstrated that an ASC/caspase-1-dependent mechanism negatively regulates AIM2/ASC complex formation or stability in *F. novicida*-infected WT macrophages.

### The ASC/Caspase-1-dependent regulation of AIM2/ASC speck formation/stability is neither due to differences in bacterial entry/replication nor in TNF-α, type I IFN or autophagy levels

**Figure 3:**
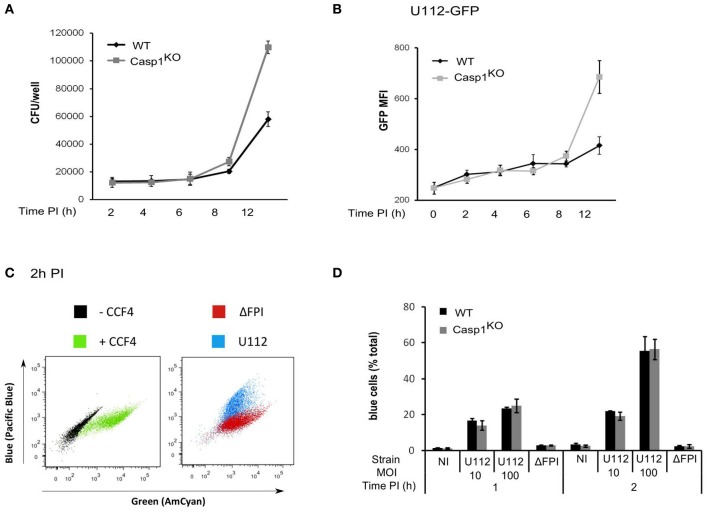
**The ASC/caspase-1-mediated negative regulation of AIM2 complex formation is not due to regulation of bacterial entry, escape or replication in the host cytosol. (A–D)** WT and Casp1^KO^ BMM, as indicated were infected with *F. novicida* U112 or ΔFPI mutant at a MOI of 10 for the indicated time. **(A)** Intracellular bacteria were numerated by CFU assay. **(B)** Mean fluorescence intensity (MFI) of intracellular GFP-expressing *F. novicida* was determined by FACS. **(C,D)** At the indicated time PI, macrophages were incubated with CCF4 for 1 h and analyzed by flow cytometry in presence of propidium iodide. Vacuolar escape in propidium iodide negative cells is revealed by the blue fluorescence emitted by the CCF4 product following cleavage by *F. novicida* β-lactamase. **(C)** Representative FACS plots overlays of uninfected WT macrophages loaded or not with CCF4 (left panel) or infected with either U112 (blue) or the Δ FPI mutant (red) and loaded with CCF4 are shown. **(D)** The proportion of blue cells was determined at the indicated time post-infection. One experiment representative of two **(C,D)** or three independent experiments **(A,B)** is shown (mean and standard deviation of triplicates are shown).

Activation of the AIM2 inflammasome upon *F. novicida* infection is dependent on the number of bacteria invading the macrophages and on the ability of the bacteria to escape into the cytosol and to replicate within macrophages (Mariathasan et al., 2005). One possible explanation for the faster AIM2/ASC speck formation occurring in Casp1^KO^ and ASC^KO^ as compared to WT macrophages could be that the level of cell invasion, escape into the cytosol or replication would be higher in Casp1^KO^ and ASC^KO^ macrophages as compared to WT macrophages. However, bacterial entry was identical between WT and Casp1^KO^ macrophages macrophages as determined by CFU assay (Figure 3A) or flow cytometry using GFP-expressing *F. novicida* (Figure 3B). To quantify *F. novicida* escape into the host cytosol, we used a technique recently used by Enninga and collaborators to monitor *Shigella flexneri* vacuolar rupture in epithelial cells (Nothelfer et al., 2011). Briefly, macrophages were loaded with CCF4, a fluorescence resonance energy transfer (FRET) probe retained within the host cytosol following the action of host cytosolic esterases. When cleaved by β-lactamase, the CCF4 FRET is lost leading upon excitation at 405 nm to a shift of fluorescence from green (535nm) to blue (450nm). *F. novicida* naturally express a β-lactamase able to cleave the CCF4 substrate (Broms et al., 2012). Its escape into the host cytosol is thus associated with a shift of the CCF4 probe from green to blue. Using this technique, we observed as previously demonstrated by others (Lindgren et al., 2004) that *F. novicida* escaped into the host cytosol in a FPI-dependent manner (Figure 3C). Furthermore, the kinetics of escape into the macrophages cytosol were identical in Casp1^KO^ and WT macrophages (Figure 3D). Similarly, during the time frame of the AIM2/ASC complex regulatory mechanism (0-6 h PI), intracellular bacterial growth was similar between WT and Casp1^KO^ macrophages (Figures 3A,B). After 6 h PI, intracellular bacterial replication was faster in Casp1^KO^ than in WT macrophages likely due to the pyroptosis of WT macrophages associated with the loss of the bacterial replicative niche. Altogether, these results indicated that the faster AIM2 specks formation kinetic observed in ASC^KO^ and Casp1^KO^ macrophages as compared to WT macrophages was not due to a lower level of invasion, escape or replication into the host cytosol in the latter cells.

**Figure 4:**
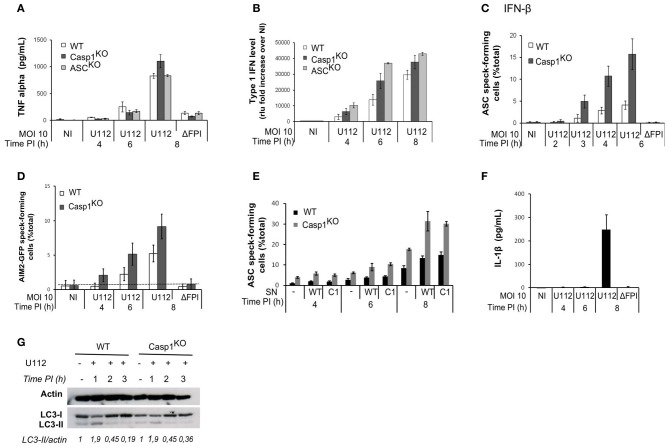
**The ASC/caspase-1-mediated negative regulation of AIM2 complex formation is not due to regulation of TNF-α, IFN-β, secreted factors or autophagy levels.** TNF-α **(A)** and type I IFN **(B)** levels in the supernatant of infected macrophages were determined by ELISA and a luciferase-based bioassay, respectively. **(C)** Macrophages were prestimulated with IFN-β (100u/ml) for 3 h before infection. ASC speck-containing macrophages were scored by immunofluorescence. **(D)** AIM2-GFP-transduced macrophages were scored for AIM2-GFP speck by fluorescence microscopy. **(E)** ASC speck-forming cells were scored by immunofluorescence in macrophages incubated 1 h PI with supernatant (SN) from WT or Casp1^KO^ BMM infected with U112 for 4 h. **(F)** IL-1β in the supernatant of WT macrophages was quantified by ELISA at the indicated time post-infection. **(G)** Immunoblot analysis of actin, LC3-I and LC3-II levels. Bands quantification was performed using ImageJ software. The numbers below the lanes were calculated by dividing the ratio of induced LC3-II to induced actin by the ratio of basal LC3-II to basal actin (band intensities). **(A–F)** One experiment representative of three independent experiments is shown (mean and standard deviation of triplicates are shown).

The NLRP3 inflammasome is positively regulated by NF-κB-activating receptors (Bauernfeind et al., 2009). Similarly, AIM2 inflammasome activation upon *F. novicida* infection proceeds faster in macrophages primed with TLR-ligands and is delayed in TLR2 and Myd88-deficient macrophages (Henry et al., 2007; Jones and Weiss, 2011). The levels of the NF-κB-dependent cytokine TNF-α were similar between WT, ASC^KO^ and Casp1^KO^ macrophages (Figure 4A). This result suggested that the delayed AIM2/ASC complex formation observed in WT macrophages was not due to a decreased NF-κB signaling.

Activation of the AIM2 inflammasome during *F. novicida* infection is dependent on type I IFN signaling (Henry et al., 2007; Cole et al., 2008; Fernandes-Alnemri et al., 2010; Jones et al., 2010). AIM2 mRNA transcripts levels were identical in uninfected WT, Casp1^KO^, and ASC^KO^ macrophages (not shown). AIM2 is an IFN-inducible protein (Burckstummer et al., 2009; Veeranki et al., 2011) AIM2-deficient macrophages have been described to secrete higher levels of IFN-β than WT macrophages upon *F. novicida* infection (Hornung et al., 2009; Fernandes-Alnemri et al., 2010). Accordingly, we observed a higher level of type I IFN secretion in Casp1^KO^ and ASC^KO^ macrophages than in WT macrophages (Figure 4B). In order to investigate if this increased type I IFN observed in inflammasome-deficient macrophages was responsible for the faster AIM2/ASC complex formation, we primed macrophages with IFN-β, 3 h before infection. IFN-β priming accelerated the kinetics of ASC speck formation in WT macrophages (Figure 4C). However, the kinetics was also accelerated in Casp1^KO^ macrophages and ASC specks were still observed earlier in Casp1^KO^ macrophages as compared to WT macrophages. Furthermore, upon ectopic expression of AIM2-GFP under a constitutive promoter, we still observed a faster kinetics of AIM2-GFP specks formation in Casp1^KO^ macrophages as compared to WT macrophages (Figure 4D). These results indicated that the difference in AIM2 speck formation could not be explained by a difference in type I IFN release or by IFN-mediated regulation of AIM2 levels.

Other cytokines apart from type I IFN are differentially secreted between WT and ASC^KO^/Casp1^KO^ macrophages (e.g., IL-1β and IL-18). Cytokines secreted in the supernatant of infected WT or Casp1^KO^ macrophages could thus be responsible for the difference in speck numbers. To investigate this possibility, we incubated macrophages with supernatant from infected WT or Casp1^KO^ BMM and quantified ASC specks upon infection. As expected the presence of supernatant from infected macrophages increased the number of ASC speck-forming cells (Figure 4E-compare at 8h PI, macrophages incubated with supernatant from either WT or Casp1^KO^ macrophages with macrophages not incubated with supernatant). However, we did not detect any differences in the ability of WT or Casp1^KO^ supernatant to increase the number of ASC specks-containing cells. Furthermore, irrespective of the origin of the supernatant, we still detected a difference between WT and Casp1^KO^ macrophages in the number of ASC-specks forming cells (Figure 4E) indicating that secreted factors could not account for the early difference in the frequency of ASC specks between WT and Casp1^KO^ macrophages. Accordingly, we did not detect IL-1β in the cell supernatant of WT infected macrophages before 8h PI (Figure 4F).

The autophagy negatively regulates inflammasome responses by dampening ROS production (Saitoh et al., 2008; Nakahira et al., 2011) and by targeting ubiquitinated inflammasome and cytosolic bacteria for destruction (Chong et al., 2012; Shi et al., 2012). We observed an increase of the autophagic process as monitored by the conversion of LC3-I to LC3-II at 1 h PI (Figure 4G). However, the autophagic process rapidly went back to its basal level. Furthermore, we did not observe any differences between WT and Casp1^KO^ macrophages at any of the time points considered. This result suggested that the autophagic process was not involved in the negative regulation of AIM2/ASC speck formation.

### Caspase-1 catalytic activity and processing are required to regulate ASC speck formation

**Figure 5:**
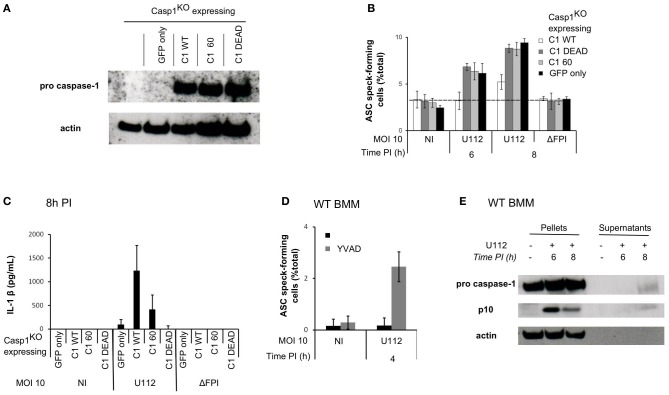
**Sublytic caspase-1 activity and processing are required to regulate ASC complex formation during *F. novicida* infection. (A–C)** Casp1^KO^ myeloid progenitors were transduced with constructs encoding the following proteins: Casp1_WT_, Casp1_DEAD_ (catalytic mutant), C60 (autoproteolytic mutant) or GFP only. **(A)** Immunoblots for caspase-1 p20 and β-actin show similar level of caspase-1 expression in macrophages transduced with the different caspase-1-encoding constructs. **(B)** Following transduction and differentiation in macrophages, cells were sorted based on GFP-expression and infected with *F. novicida* at a MOI of 10:1. ASC speck-forming cells were scored by immunofluorescence. **(C)** IL-1 β secretion in the supernatant of transduced macrophages was determined by ELISA at 8 h PI. **(D)** WT macrophages were treated with z-YVAD-CMK at 1h post-infection, ASC specks forming cells were scored by immunofluorescence. **(E)** Immunoblots for caspase-1 p10 subunit and β-actin were performed on cell lysates and on the corresponding supernatants. One experiment representative of two **(A–C)** or more than two **(D,E)** independent experiments is shown (mean and standard deviation of triplicates are shown).

Caspase-1 is a cysteine protease expressed as a procaspase within the cell. Upon recruitment to an inflammasome complex, caspase-1 is activated and processed into a mature caspase-1 formed of p10 and p20 subunits. Mutation of the cysteine in the catalytic site results in an inactive caspase unable to cleave any substrates. However, caspase-1 has scaffolding activities independently of its enzymatic activity (Lamkanfi et al., 2004). To investigate the role of caspase-1 catalytic activity in the regulation of ASC speck formation, we infected Casp1^KO^ macrophages ectopically expressing wild-type caspase-1 (Casp1_WT_) or a catalytically inactive caspase-1 mutant (Casp1_DEAD_) (Figure 5A) (Broz et al., 2010). Importantly, Casp1_WT_ ectopic expression delayed ASC speck formation (Figure 5B) demonstrating that Casp1^KO^ macrophages could be complemented by expression of WT caspase-1. In contrast, expression of Casp1_DEAD_ did not complement Casp1^KO^ macrophages indicating that the delay in ASC speck formation in WT macrophages required caspase-1 catalytic activity. As expected, ectopic expression of Casp1_WT_ restored IL-1β secretion in Casp1^KO^ macrophages, while ectopic expression of Casp1_DEAD_ did not (Figure 5C). The (p10)_2_(p20)_2_ processed caspase-1 has long been considered as the only active caspase-1. Recently, Broz et al. generated uncleavable procaspase-1 mutants and evidenced that caspase-1 zymogen, within certain inflammasome structures, could trigger cell death without being processed (Broz et al., 2010). To investigate the role of caspase-1 processing in the regulation of ASC speck formation, we infected Casp1^KO^ macrophages ectopically expressing uncleavable caspase-1 [C60 construct (Broz et al., 2010)]. In contrast to the ectopic expression of Casp1_WT_, expression of the autoproteolytic mutant C60 did not delay ASC speck formation (Figure 5B). Altogether, these results indicated that both the catalytic activity of caspase-1 and its cleavage were required to negatively control ASC speck formation or stability. To confirm these results using a different technique, we treated WT macrophages with the caspase-1 inhibitor zYVAD-FMK. YVAD treatment led to the appearance of ASC specks in WT macrophages as early as 4 h PI (Figure 5D), thus confirming the results obtained by genetic complementation of Casp1^KO^ macrophages. As reported by others (Broz et al., 2010), this inhibitor only partially reverted *F. novicida*-mediated cell death (45% cell death in untreated WT macrophages infected with U112 at a MOI of 100:1 for 6 h as compared to 26 % cell death in zYVAD-FMK-treated macrophages). We could not test the pan-caspase inhibitor zVAD-FMK due to its toxicity on primary macrophages (Martinet et al., 2006) possibly due to the activation of RIP-3-mediated cell death (Kaiser et al., 2011).

### Caspase-1 activity is observed in *F. novicida*-infected macrophages before pyroptosis and could be responsible for the negative feedback loop

Caspase-1 activity was required to delay AIM2/ASC speck formation suggesting that caspase-1 could be activated within infected macrophages without triggering an immediate cell death. A sublytic caspase-1 activity (activity observed in absence of cell death) has been previously described in epithelial cells (Gurcel et al., 2006) but it is still very poorly documented in primary macrophages. Due to the high level of cross-reactivity of the caspase-1 fluorescent probe FLICA_casp1_ with other caspases activated in a caspase-1-independent manner (Pierini et al., 2012; Puri et al., 2012), we were unable to monitor caspase-1 activity at the single cell level. To investigate the presence of sublytic caspase-1 activity, we thus analyzed caspase-1 processing by western blot at 6 h PI (Figure 5E), i.e., before the detection of cell death (Figures 2A–C). We could not detect any processed caspase-1 in the cell supernatant at this time point. However, we observed the appearance of the p10 form in the cell lysate of WT infected macrophages at 6 h PI suggesting that caspase-1 activation and processing can occur within WT macrophages in absence of pyroptosis. In contrast at 8 h PI, when pyroptosis has started to happen, we observed caspase-1 p10 form both in the cell lysate and in the cell supernatant. Together with the ectopic expression of caspase-1 DEAD and C60 mutants and the use of the YVAD inhibitor, this result suggested that sublytic caspase-1 activation and processing negatively regulated in an ASC-dependent manner AIM2 speck formation in *F. novicida*-infected macrophages.

### ASC speck forms earlier in casp1^KO^ macrophages as compared to WT macrophages upon DNA transfection but not upon nigericin treatment or *legionella pneumophila* infection

**Figure 6:**
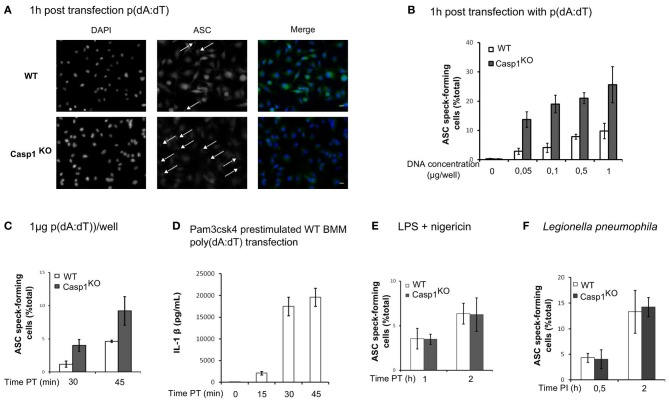
**ASC/caspase-1 negative regulation is specific of AIM2 inflammasome. (A–D)** BMM were transfected with p(dA:dT). **(A)** Endogenous ASC was detected by immunofluorescence (scale bar: 10 μm). ASC speck-forming cells were scored after transfection of a large range of p(dA:dT) concentrations **(B)**, at different times post transfection with 1 μg of p(dA:dT) per well **(C)**, after nigericin (10 μM) treatment of LPS-prestimulated BMM **(E)** or following *Legionella pneumophila* infection (MOI 10) **(F)**. **(D)** IL-1β concentration in the supernatant of macrophages prestimulated with Pam3CSK4 was determined at the indicated time post-transfection by ELISA. One experiment representative of two **(E,F)** or more than two **(A–D)** independent experiments is shown (mean and standard deviation are shown).

AIM2 inflammasome activation upon *F. novicida* infection is due to the release of bacterial DNA into the host cytosol (Fernandes-Alnemri et al., 2010; Jones et al., 2010). Similarly, direct transfection of synthetic DNA into the cytosol can activate the AIM2 inflammasome. To investigate if caspase-1-mediated regulation of ASC speck formation was specific for *F. novicida* infection or a general property of the AIM2 inflammasome, we transfected WT and Casp1^KO^ macrophages with p(dA:dT). Although the differences are to be interpreted with caution due to cell death occurring very rapidly in WT macrophages (data not shown), we observed much more ASC-specks in Casp1^KO^ as compared to WT macrophages (Figure 6A). The greater number of ASC-specks forming Casp1^KO^ macrophages was observed upon transfection of a large range of p(dA:dT) concentrations (Figure 6B) and was detected as soon as 30 min post-transfection (Figure 6C). At this time point, three times more ASC specks were numbered in Casp1^KO^ macrophages as compared to WT macrophages. The short period required to visualize ASC specks was consistent with the kinetics of IL-1β release observed in WT macrophage following p(dA:dT) transfection (Figure 6D). This observation suggested that the regulation of AIM2/ASC speck formation by active caspase-1 was a general phenomenon affecting the AIM2 inflammasome independently of the source of DNA recognized. We next investigated whether this caspase-1-mediated regulation was also affecting ASC-speck formation upon activation of the NLRP3 inflammasome with LPS plus nigericin (Mariathasan et al., 2006) or upon activation of the Naip5/Ipaf inflammasome with *Legionella pneumophila* (Lightfield et al., 2008). As presented in Figures 6E,F, we did not notice any significant regulation of ASC-speck formation using these stimuli suggesting that the ASC/caspase-1 regulatory pathway described here was specific of the AIM2 inflammasome pathway.

## Discussion

Inflammation is a key process to fight microbial infections but is also implicated in severe acute immune disorders and numerous auto-inflammatory syndromes. Negative feedback loops have been described in TLRs, NLRs and RLRs signaling pathways (Coll and O'Neill, 2010) illustrating the importance of such pathways to limit, control and resolve inflammation. Here, we describe an AIM2 inflammasome negative feedback loop affecting the kinetics of formation of visible AIM2/ASC specks upon *F. novicida* infection or p(dA:dT) transfection. We demonstrate that this regulatory mechanism requires caspase-1 catalytic activity and takes place before caspase-1-mediated pyroptosis.

The lower number of specks visible by immunofluorescence and of ASC dimers, trimers detected by western blot following chemical cross-linking in WT macrophages could be due either to a delay in speck formation or to a lower stability/higher turn-over of the complexes formed in WT macrophages. Further investigations are required to discriminate the two possibilities but the formation of an unstable complex might explain the presence of active caspase-1 detected at 6 h PI in WT macrophages.

The role of caspase-1 in triggering pyroptosis and in processing proIL-1β and proIL-18 into mature cytokines is well-established. However, the sublytic roles of caspase-1 are still largely uncharacterized. Caspase-1-mediated speck formation regulation occurs well before caspase-1-mediated cell death. In support of a sublytic level of active caspase-1, we detected caspase-1 p10 form in the cell lysate at early times PI. Sublytic roles of caspase-1 have been identified in fibroblasts exposed to pore-forming toxins (Gurcel et al., 2006), and in macrophages infected with *L. pneumophila* at a low MOI (Amer et al., 2006). The mechanisms balancing caspase-1 sublytic and lytic activities remain to be deciphered.

The mechanism of AIM2 inflammasome activation has been recently uncovered thanks to the characterization of the crystal structure of its HIN domain in complex with dsDNA (Jin et al., 2012). As previously described (Jones et al., 2010), AIM2 speck forms in ASC-deficient macrophages. The relevance of such a complex in inflammasome-deficient cells is still unknown. Two mechanisms might explain AIM2 speck formation. First DNA could serve as an oligomerization platform for AIM2. Second, the pyrin domain of AIM2 has an intrinsic ability to oligomerize/aggregate as seen upon ectopic expression (Hornung et al., 2009; Jin et al., 2012). The regulation of AIM2 oligomerization is still unclear. In fact, the regulation of the different inflammasome pathways is still largely unknown [for a recent review see Rathinam et al. (2012)]. Recently, autophagy has been described as a specific negative regulator of the AIM2 inflammasome. Autophagy is activated following AIM2 speck formation, which leads to the removal of AIM2 specks. This activity is independent of ASC and caspase-1 (Shi et al., 2012) suggesting that the negative feedback loop that we describe in this study does not share the same mechanisms of action. Accordingly, we did not observe any differences in autophagy levels between WT and Casp1^KO^ BMM suggesting that the ASC/caspase-1-mediated negative feedback loop acts independently of autophagy.

Using ectopic expression of caspase-1 catalytic mutants in Casp1^KO^ macrophages or zYVAD-FMK treatment of WT macrophages, we demonstrated that caspase-1 catalytic activity was required to affect ASC specks formation. The requirement for caspase-1 catalytic site to regulate AIM2 inflammasome kinetics indicates that a caspase-1 substrate is involved in the feedback loop mechanism. Our favored hypothesis is that such a caspase-1 substrate acts on AIM2 inflammasome formation or stability to delay the full activation of this complex in WT cells. Such a post-translational mechanism is consistent with the very fast kinetics of the regulation observed upon p(dA:dT) transfection (Figure 5C), which is mirroring IL-1β release kinetics (Figure 5D). We did not detect any caspase-1-dependent cleavage of AIM2 (not shown). However, regulation of AIM2 complex could be mediated by proteins (de)stabilizing the AIM2 complex or competing with AIM2 for DNA binding. ZAPS is a protein promoting RIG-I polymerization (Hayakawa et al., 2011), it remains to be investigated whether such an AIM2 regulatory protein exists. Furthermore, AIM2 belongs to a large family of HIN-domain containing proteins or AIM2-like receptors, which has been recently characterized (Brunette et al., 2012). Some of these ALRs (Roberts et al., 2009; Veeranki et al., 2011) have been reported to act as negative regulators of the AIM2 pathway and it remains to be seen whether they participate in the feedback loop mechanism. Alternatively, AIM2 inflammasome regulation might reflect regulation at the level of the DNA ligand. Caspase-activated DNAses (Torriglia and Lepretre, 2009) could regulate AIM2 ligand availability and thus impact inflammasome activation. The identification of specific caspase-1 substrate remains a challenge. Indeed, caspase-1 has a promiscuous activity (Walsh et al., 2011) and its recruitment to a complex (such as the AIM2 complex) might be more important to determine locally the substrates repertoire than specific peptidic sequences.

While ASC speck formation kinetics is accelerated in Casp1^KO^ macrophages upon engagement of AIM2 by *F. novicida* DNA or p(dA:dT), we did not observe any significant impact of caspase-1 on *L. pneumophila*-mediated or nigericin-mediated ASC speck formation. This result indicates that the caspase-1-mediated feedback loop is specific for the AIM2 inflammasome. Such specificity is consistent with the regulation of AIM2 speck formation occurring in ASC^KO^ macrophages, which pinpoints the regulatory step upstream or at the level of AIM2 protein. ASC acts as a negative modulator of the Naip5/NLRC4/caspase-1-mediated cell death during *L. pneumophila* infection (Case and Roy, 2011). Furthermore, caspase-1 triggers membrane repair and promotes survival in cells intoxicated with bacterial pore-forming toxins (Gurcel et al., 2006). Depending of the inflammasome pathway considered, different regulatory mechanisms might thus be at play to dampen or resolve inflammasome activation. Nevertheless, negative feedback loops implicating inflammasome components are emerging as a common theme. We did not manage to decipher the molecular mechanisms underlying this negative feedback loop. Identification of the molecular players and particularly the putative caspase-1 substrate discussed above should allow the investigation of the relevance of this regulatory pathway in WT cells and animals. Mutations in genes encoding proteins involved in this regulation might contribute to lower the cytosolic DNA detection threshold required for caspase-1 activation possibly leading to auto-inflammatory syndromes such as psoriasis, which is associated with AIM2 inflammasome activation (Dombrowski et al., 2011).

### Conflict of interest statement

The authors declare that the research was conducted in the absence of any commercial or financial relationships that could be construed as a potential conflict of interest.
